# Impact of Water, Sanitation, and Hygiene Interventions on Improving Health Outcomes among School Children

**DOI:** 10.1155/2013/984626

**Published:** 2013-12-28

**Authors:** Ashish Joshi, Chioma Amadi

**Affiliations:** Center for Global Health and Development, College of Public Health, UNMC, 984355 Medical Center, Omaha, NE 68198-4355, USA

## Abstract

*Purpose*. This review was done to explore the impact of water treatment, hygiene, and sanitary interventions on improving child health outcomes such as absenteeism, infections, knowledge, attitudes, and practices and adoption of point-of-use water treatment. *Methods*. A literature search was conducted using the databases PubMed and Google scholar for studies published between 2009 and 2012 and focusing on the effects of access to safe water, hand washing facilities, and hygiene education among school-age children. Studies included were those that documented the provision of water and sanitation in schools for children less than 18 years of age, interventions which assessed WASH practices, and English-language, full-text peer reviewed papers. *Results*. Fifteen studies were included in the final analysis. 73% (*n* = 11) of the studies were conducted in developing countries and were rural based (53%, *n* = 8). The child's age, gender, grade level, socioeconomic index, access to hygiene and sanitary facilities, and prior knowledge of hygiene practices were significantly associated with the outcomes. Nutrition practices which are key factors associated with the outcomes were rarely assessed. *Conclusion*. Further research is required to assess the long-term impact of such interventions in different settings.

## 1. Introduction

The poor access to water supply is a prevalent issue in over 850 million people worldwide with over 2.5 billion limited by access to sanitation facilities [1]. The global burden of disease and mortality rates could be reduced by about 9.1% and 6.3%, respectively, if rapid success is attained in facilitating access to water, sanitation, and hygiene facilities [2]. A large proportion of these diseases are related to diarrhea incidences which contribute to the mortality rate of about 1.9 million and new diarrhea cases estimated at 4 billion annually especially among children under five years old [3]. Developing countries account for around 19% of those mortality rates [3]. The World Health Statistics review done in 2009 showed that the highest case fatality rates due to diarrheal incidences occurred in India with over 386,000 diarrheal deaths [1]. High mortality rates of about 13.9% are still attributed to diarrheal deaths in Egypt among children less than five years old irrespective of the recent reduction in child mortality rates [1]. The leading cause of infant mortality and health-related expenditures has been attributed to diarrheal incidences among children in Indonesia [4]. Diarrheal diseases are also the third cause responsible for increased morbidity rates in all age groups in Indonesia [4].

Previous literature has shown considerable studies regarding the effects of lack of appropriate water facilities, hand washing, and hygiene practices on child health outcomes. Impaired cognitive learning and learning performance are long-term outcomes of the negative effects of infections such as diarrhea, worm infestations, and dehydrations which are largely attributed to poor water, sanitation, and hygiene conditions [5]. Diarrheal incidences in children during their first few years of life have been shown to limit their growth by about 8 cm and cause an IQ point reduction when they progress to about 7 or 8 years of age [6]. Studies have shown that about 75% of all school absences are illness related [7]. Information regarding absenteeism from middle and higher income countries has shown that poor academic and social development, high dropout rates, and reduced learning performance are attributed to school absence in children [8–11].

“Attendance is a strong predictive factor of academic success for elementary school pupils” [12]. Absenteeism due to illness has been shown to be reduced by implementation of mandatory hand hygiene and sanitary procedures based on the results of previous interventions [13]. The availability and utilization of alcohol-based sanitizers in schools have also been shown to reduce absenteeism by about 20–50% [14–17]. A hand hygiene intervention in two public elementary schools in Chicago involving instructions in hand hygiene practices and provision of hand hygiene facilities significantly reduced absenteeism among students in prekindergarten to the eighth grade (ages 4–14) [7].

There have been considerable studies that have examined the effect of water treatment, hygiene, and sanitary practices on reducing absenteeism, diarrhea prevalence, and acute respiratory infections in school-age children. However, limited research has been done to evaluate the effectiveness of water, sanitation, and hygiene practices through randomized controlled clinical trials to gauge the long-term impact of these interventions on improving child health outcomes. The objective of the study is to examine and describe the gaps in the existing water, sanitation, and hygiene interventions to improve child health outcomes such as acute respiratory infections, diarrheal episodes, and absenteeism in various settings.

## 2. Methods

A search was conducted in January 2013 using the scientific databases PubMed and Google scholar for studies published between 2009 and 2012 and focusing on the effects of access to safe water, hand washing facilities, and hygiene education among school-age children. Key words were used either in single form or in combination and included “Water access and Waterborne illnesses,” “School enrollment and Hygiene education,” “Hand washing facilities and absenteeism,” and “Hygiene education and children.” Studies included were those that documented the provision of water and sanitation in schools for children less than 18 years of age, interventions which assessed the impact of WASH practices, and English-language, full-text peer reviewed papers. Studies which did not have a school-based component in assessing WASH practices were excluded. A secondary search was also done to review the references of the articles included in the final analysis.

## 3. Results

**Figure 1:**
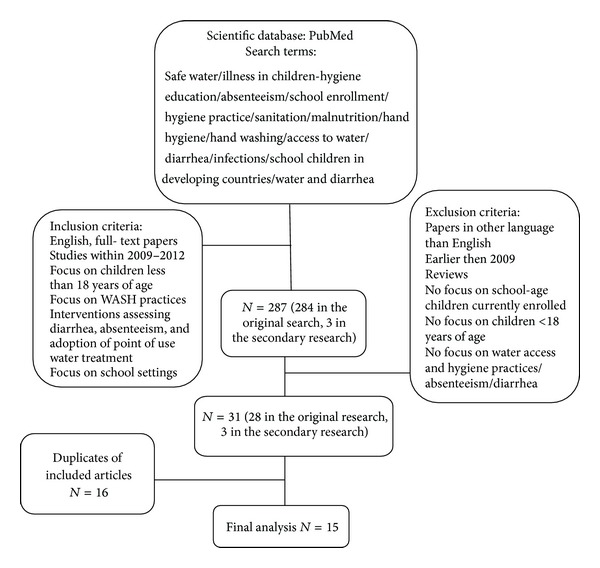
Flow chart showing procedures undertaken in article selection.

The initial search yielded 287 studies including the secondary research. This was reduced to 15 studies after the exclusion criteria were applied and duplicates removed (Figure 1). 40% (*n* = 6) of the studies were published in 2011 and 33% (*n* = 5) in 2012. 73% (*n* = 11) of the studies were conducted in developing countries including India, Kenya, and Egypt and were rural based (53%, *n* = 8). 60% (*n* = 9) of the studies incorporated an educational component in the interventions assessing the impact of water treatment and sanitation hygiene and this was shown to facilitate the success of those studies. The outcomes assessed were reducing illness-related absenteeism, gastro-intestinal, and respiratory infections and adoption of point-of-use water treatment in children. Hygiene and sanitation interventions have had considerable impact on reducing diarrhea and absenteeism rates in school-age children.

## 4. Variable Extraction

### 4.1. Sociodemographic Factors

- Age/grade level: comparisons of the effectiveness of WASH interventions were reported for some of the studies stratified by various age categories and measured in years.
- Gender: the gender of the children in the various studies was noted to observe if there were any differences in the effectiveness of the interventions.
- Socioeconomic status/household income: information on socioeconomic status was abstracted from the articles to observe its significance towards promoting access to water facilities and improving hygiene practices in children.
- Parental literacy: it was assessed to monitor the effect of role-modeling behaviors in children towards imbibing knowledge, attitudes, and practices of sanitation and hygiene.

### 4.2. Research Methods

- Study design: information was gathered to determine the types of studies that were performed including randomized controlled trials, cross-sectional studies, cohort studies, and case series.
- Study location: information was gathered about the locations where the studies were conducted.
- Study duration: the period of the study was also recorded.
- Sample size of the populations: it was also recorded.

### 4.3. Intervention

- Knowledge: knowledge, as an educational component of the various studies reviewed, was assessed to observe their effects on sustained impact of the interventions. Comparisons were also made to interventions that did not utilize this component to weigh its effectiveness.

### 4.4. Outcomes

- The outcomes assessed included absenteeism, diarrhea, and malnutrition in children.

### 4.5. Sociodemographic Factors

#### 4.5.1. Ages of Children Studied

The commonly studied age groups of children studied fell between 5 years and 16 years (53%, *n* = 8). 13% (*n* = 2) of the studies focused on children less than 5 years while 26% (*n* = 4) of the studies did not specify the age groups of the children.

### 4.6. Research Methods

#### 4.6.1. Study Locations

**Figure 2:**
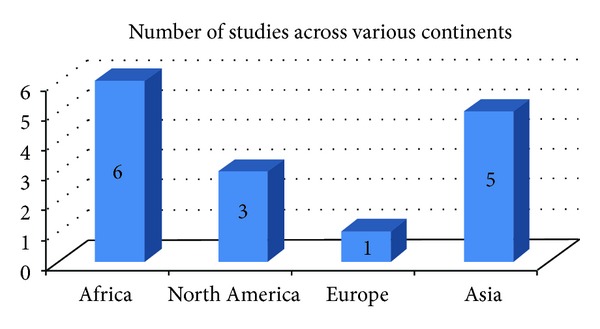
Number of studies across various continents.

40% (*n* = 2) of the studies were conducted in Africa (Figure 2). Others were conducted in Asia (33%, *n* = 5), North America (20%, *n* = 3), and Europe (6%, *n* = 1).

### 4.7. Study Design

More than half (53%, *n* = 8) of the studies were randomized clinical trials while 47% (*n* = 7) were cross-sectional studies. Only one study was a case series (*n* = 1).

### 4.8. Study Setting: Rural/Urban/Both

100% (*n* = 15) of the studies reviewed were school based, conducted in primary/middle/elementary schools, with one study incorporating a community component. More than half of the studies were done in rural settings (53%, *n* = 8). 33.3% (*n* = 2) of the studies done in Africa were urban based, 80% (*n* = 4) of the studies done in Asia were rural based, and 100% (*n* = 3) of the studies done in North America were urban based.

### 4.9. Sample Sizes

The total sample size ranged from 76 to 44451 children. 53% (*n* = 8) of the studies had sample sizes ranging between 500 and 1000.

### 4.10. Sources of Data Collection

**Figure 3:**
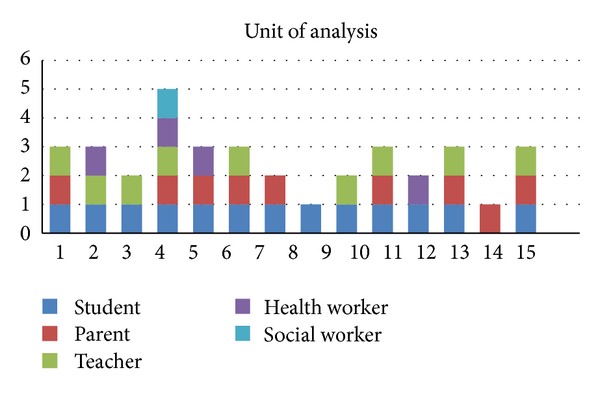
Chart showing the different units of analysis.

The data collection process was carried out using surveys at several levels including students, parents, teachers, health workers, social workers, or a combination of them (Figure 3).

### 4.11. Variables Assessed

Several variable categories were gathered based on the literature review of the articles included in the final analysis. These include the following.

### 4.12. Sociodemographics

*(i) Age.* 80% (*n* = 12) of the studies reviewed assessed the ages of the children in relation to the outcomes. 53% (*n* = 8) of the studies included children with age groups between 5 years and 16 years. 13% (*n* = 2) of the studies reviewed involved children less than 5 years of age, while 26.6% (*n* = 4) of the studies did not specify the ages of the children. 27% (*n* = 3) of the studies which assessed age found a significant association with the outcomes. Results from a study that examined the prevalence of helminthic infections in children showed that the age of children was a significant factor in the risk of helminthic and protozoan infections [18]. Older children aged 8 and 10 years were less prone to infections by helminthes and intestinal protozoa, unlike the younger children between 7 and 8 years who had very high infection rates [18]. However, in another study, the infection rates were significantly associated with older children between 8–10 years old [19]. The ages of the children were also significantly associated with latrine use especially in the 8–10 age groups [10].

*(ii) School Grade Levels.* 86% (*n* = 13) of the studies assessed the school grade levels of the children. Majority of the children studied fell between grades ranging from pre-kindergarten to the eighth grade (86%, *n* = 13). 13.3% (*n* = 2) of the studies did not specify the grades of the children. The association between school grades and the outcomes was significant in 23% (*n* = 3) of the studies. The child health outcomes reviewed were more significantly associated with children in higher grade levels [14]. 

*(iii) Gender.* The gender of the participating children was described in majority of the studies reviewed (*n* = 12, 80%). 20% (*n* = 3) of the studies found a significant association between gender and the outcomes. Two of those studies were randomized control trials assessing the impact of water treatment and sanitation hygiene (WASH) on reducing absenteeism and diarrhea/ARI prevalence and 1 was a cross-sectional study. In the randomized controlled trials, absenteeism reduction was significant in girls [1, 20], but in the cross-sectional study, the impact was associated with the male gender. 

*(iv) Parental Literacy.* 66% of the studies assessed parental literacy (*n* = 10) and 50% of those collected data specifically on maternal education (*n* = 5). Maternal literacy was found to be insignificant in one of the studies involving the factors associated with the use of improved water sources and sanitation among children in Cambodia [20] and its effect was not specified in others studies [18, 21–24]. The significance of parental literacy on the outcomes assessed was not stated in 5 of the studies reviewed [18, 20, 21, 24, 25].

### 4.13. Socioeconomic Index

- The socioeconomic index was described in the studies reviewed to include wealth index, wealth, household income, household assets, and housing type. 60% of the studies analyzed the association between socioeconomic index and the outcomes (*n* = 9), but its significance was only stated in 3 studies [18, 20, 21]. Higher socioeconomic status (defined by housing type) was a key independent factor associated with the use of improved water sources and sanitation [20]. Results from a study showed that children belonging to households of the middle 40% which were on a higher scale had a reduced risk of being infected compared to their poorer counterparts (OR = 0.58, 95% CI: 0.38–0.90) [18]. Individuals with higher socioeconomic index generally reflected the positive outcomes of the health interventions compared to those with a lower index [21].

### 4.14. Household Variables

- Family size (26.6%, *n* = 4) and the number of female-headed households (20%, *n* = 3) were the most common household variables assessed. 13.3% (*n* = 2) of the articles showed a significant association between female-headed households and the outcomes while the remaining article did not specify its significance. 25% (*n* = 1) of the studies that assessed family size showed a significant association with the outcomes. The other variables assessed included distance to school from home, distance to water source, climatic season, number of people sharing bedroom/toilet with child, pupil to latrine ratio, water quantity used, and crowding index. Data on other household variables than the listed above were rarely collected in all the studies reviewed.

### 4.15. School Characteristics

- Latrine coverage in schools was the most common variable assessed (60%, *n* = 9) followed by school area/location and water source at school (13.3%, *n* = 2) each. Information gathered on school characteristics included school area/location, latrine coverage, pupil-latrine ratio, water source at school, hand washing soap on basin, means of waste disposal, and washup point after toilet. 44.4% (*n* = 4) of the studies that assessed latrine coverage showed a significant association with the outcomes while 50% (*n* = 2) of the studies that assessed school area (highlands versus lowlands) and water sources found a significant association with the outcomes. The prevalence of diarrheal infections was lowest in children from households located in mountainous regions [10]. In rural Cambodian school children, diarrheal incidences were largely reduced in households which utilized improved water sources and sanitation facilities [14].

### 4.16. Environmental Characteristics

- The major data on environmental characteristics collected in most studies were those regarding drinking water sources in households (46.6%, *n* = 7) and obtaining water samples (26.6%, *n* = 4). 42.9% (*n* = 3) of the studies that assessed drinking water sources found a significant association with the outcome. Improved water sources including public wells and stand pipes were significantly associated with positive health outcomes [10]. Chlorine treatment was not significantly associated with improved child health outcomes in a randomized controlled trial [9]. Other data assessed included water source contamination/water quality and water access (availability). Data regarding water source contamination and water access were sparsely recorded.

### 4.17. Nutrition Practice

- 20% (*n* = 3) of the studies assessed the association between nutrition practices and the outcomes. The variables collected included: food handling food preparation, food consumption, acute malnutrition, breast feeding, and meat consumption. The studies did not state the association between nutrition practices and the outcomes.

Other data collected in the studies reviewed included nasal swab samples (for influenza testing), age of respondents, crowding index, sewage spillage, and contaminated fomites. Each variable in the particular category has been described in detail below.

*(i) Knowledge, Attitudes, and Practices.* The most commonly assessed variables in the studies reviewed include hand washing (66.6%, *n* = 10), latrine coverage (60%, *n* = 9), sanitation practices (53.3%, *n* = 8), prior knowledge, and use of soap (46.6%, *n* = 7). Majority of the studies showed a significant association between these variables and the outcomes (see Table 1). A reduced risk of parasitic infections was observed in children with substantial knowledge regarding hygiene and sanitation practices (AOR 0.78, CI 0.56–1.09) [5]. Their knowledge was also reflected in their clean clothing (AOR 1.62, CI 1.14–2.29).

## 5. Interventions

The interventions included in the final analysis were in single form or in combination. These are outlined below.

### 5.1. Single Interventions

#### 5.1.1. Hygiene Practices

There was only one study that examined the impact of hygiene practices on absenteeism due to infectious illness among pupils in elementary schools. The study was conducted in Denmark. Two schools comprising 652 students were randomized into an intervention and a control group. The students at the intervention school were required to wash their hands before the first lesson, before lunch, and before going home while those at the control school continued their usual hand washing practices. The rates of absenteeism for the students in the intervention school were significantly reduced compared with those in the control school (*P* = 0.002). The effects of reduced absenteeism were more prominent on female students (1.05, 95% CI 0.90–1.22) compared to their male counterparts (0.87, 95% CI = 0.72–1.05) [13].

### 5.2. Combined Interventions

#### 5.2.1. Hygiene Education and Access to Hygiene Resources

There were 4 studies that examined the impact of the combination of hand hygiene instructions and provision of hygiene facilities. They included both US- and non-US-based studies conducted in Texas, Chicago, Egypt, and Pittsburgh.

The study conducted in North Texas was geared towards controlling a Shigella outbreak in an elementary and middle school [26]. Installing liquid soap in dispensers in student restrooms followed by sustained instruction in hand washing and monitoring of hygiene practices among students was performed. The results showed that appropriate soap supplies and repeated instruction in hand washing and its monitoring were needed to control the Shigella outbreak. The study conducted in Chicago assessed the role of hand hygiene instruction in decreasing illness-related absenteeism in two public elementary schools during the peak flu season [7]. Classrooms were systematically assigned to an intervention or control group by grade. Hand hygiene facilities were made available to all students. Students in the intervention group also received short repetitive instruction in hand hygiene every two months. The results of the study showed higher rates of attendance among students that received hand hygiene instruction during the flu season (*P* = 0.002, *P* < 0.001, resp.).

The study conducted in Egypt assessed the effects of hand hygiene campaigns on the incidence of laboratory-confirmed influenza and absenteeism in school children [27]. The study design was a randomized controlled trial involving 60 elementary schools. Children in the intervention schools were required to wash hands twice daily and health messages were provided through entertainment activities. An intensive campaign to promote hand hygiene was launched in the intervention schools to raise the awareness of students, teachers, nurses, and parents; it required students to wash their hands at least twice during the school day for 45 seconds, followed by proper rinsing and drying with a clean cloth towel. The results showed a significant reduction in illness-related absenteeism, infections rates including diarrhea, conjunctivitis, and influenza by the following frequencies: 40%, 30%, 67%, and 50%, respectively (*P* < 0.0001).

In another randomized controlled trial, children in 5 intervention schools received training about hand and respiratory hygiene and were provided and encouraged to use hand sanitizer regularly while the children in the other 5 schools acted as controls. Prior to implementation of the interventions, there was a 45-minute presentation at intervention schools regarding influenza and proper hand washing technique and sanitizer use. Hand sanitizer dispensers with 62% alcohol-based hand sanitizer were installed in each classroom and all major common areas of intervention schools. The children were required to utilize it several times daily including upon arrival, before and after lunch, and prior to departure was taught to students. They were also encouraged to wash hands or use additional doses of hand sanitizer as needed. The total absence episodes were significantly reduced among children in the intervention group compared to the controls, adjusted IRR 0.74 (95% CI: 0.56, 0.97).

#### 5.2.2. Water Treatment/Hygiene Practices/Hygiene Education

There were 3 randomized controlled trials that utilized a combination of water treatment and hygiene practices and education (Kenya, *n* = 2; India, *n* = 1) [21, 28, 29]. One of the prior studies done in Kenya assessed the impact of a hygiene curriculum and the installation of hand washing and drinking stations towards reducing diarrhea and acute respiratory infections in students [29]. Water treatment involved the installation of water stations utilizing a water guard solution. Hygiene education was provided through the provision of instructional materials and training for teachers and students while water stations installed were strategically located near latrines for hand washing to improve hygiene practices. The results showed a decrease in the median percentage of students with acute respiratory illness but no decrease in diarrhea among students.

In another study conducted in Kenya, the role of school children in the promotion of point-of-use water treatment and hand washing in schools and their impact on reducing pupil absenteeism rates was evaluated [28]. Water stations which utilized a flocculent/disinfectant called PuR were installed and teachers and students received training on hygiene as well as instructional books. Student absenteeism rates decreased after implementation by 26%. In another study conducted in India, the impact of a school-based safe water intervention on household adoption of point-of-use water treatment practices was assessed [21]. The intervention consisted of providing classrooms of 200 schools with a commercial water purifier, training of students and teachers, and provision of basic hygiene and water treatment information. There was no evidence that the intervention was effective in improving uptake of water treatment practices.

#### 5.2.3. Water Treatment and Hygiene Promotion (Hygiene Practices and Hygiene Education) and Sanitation

Only one study was conducted in Kenya which utilized multiple intervention components involving water treatment, hygiene promotion, and sanitation to assess their impact on pupil absence [22]. Schools were randomized to one of three study arms including water treatment and hygiene promotion (WT and HP), water treatment, hygiene promotion, and sanitation (WT, HP, and Sanitation), and a control group. The intervention provided water guard (a 1.2% chlorine based point-of-use water disinfectant for water treatment). Hygiene promotion incorporated an education component which involved a 3-day training of teachers as well as provision of hand-washing and drinking water containers while the sanitation component involved the provision of latrine facilities to the students. The addition of a sanitation component as an intervention arm resulted in a marginally significant reduction in absenteeism among the students (OR 0.47, 0.21–1.05).

## 6. Results

### 6.1. Major Outcomes Assessed

The studies reviewed assessed 4 major outcomes of the effect of water and sanitation hygiene practices in children. These included absenteeism, infections (diarrhea/acute respiratory), knowledge/attitudes/practices, and adoption of point of use water treatment (Table 2).

### 6.2. Absenteeism

Information regarding absenteeism was defined and collected differently in the various studies reviewed [13, 19, 22, 27, 30]. Absence periods were defined as “number of days absent due to a single cause, with at most 2 days of attendance or a weekend between events and was recorded based on the number of absences due to infectious causes as reported by pupils, teachers and parents” [13], or “number of absences caused by illness per 100 student-weeks and was recorded based on the number of absences due to illness as reported by parents and school records” [27], or measured using “2-week pupil reported absence” [22], “illness related absence as reported by parents” [19], or “number of absences due to illness and non-illness from both parents and teachers” [30]. One of the studies did not define absence days but absence records were ascertained from schools [28].

Five studies assessed the impact of WASH practices on reducing absenteeism. Illness-related absenteeism children constitutes about 75% of all school absences [7] and is largely attributed to respiratory and gastro-intestinal infections. Incorporating an educational component in the interventions was shown to be very effective in improving the outcomes. Access to hand hygiene instruction and hygiene facilities improved attendance at public elementary schools during the flu season [7]. Of all the studies that were geared towards reducing absenteeism, gender was significant in 33% (*n* = 2) of the studies. The benefits of hand washing were more pronounced in females with the highest rates of absenteeism [10, 13]. Some of the limitations of the existing studies were self-reporting with regard to illness incidences and compliance. Others include small sample sizes, and loss to followup.

### 6.3. Knowledge, Attitudes, and Practices

The variables assessed regarding knowledge, attitudes, and practices included knowledge of prior water treatment, use of soap/sanitizer (46%, *n* = 7), latrine coverage in household and community (60%, *n* = 9), hand washing frequency (80%, *n* = 12), sanitation practice (latrine use, 53%, *n* = 8), household water treatment, water storage practices (20%, *n* = 3), and prior knowledge of hygiene practices (20%, *n* = 3). Only 5 studies gathered information about the knowledge, attitudes, and practices of school children in relation to infections (diarrhea, intestinal protozoa, schistosomiasis) and the use of improved water sources. Data regarding drying material availability, hygiene practices (taking bath, brushing teeth, and washing hair and feet), sewage spillage, and vaccination were sparsely collected in the studied reviewed.

### 6.4. Infections

The most common infections included diarrheal and acute respiratory infections. 46% (*n* = 7) of the studies in this review assessed water access, sanitation, and hygiene practices in relation to diarrhea prevalence and other infectious disease incidences among school-age children. The risk factors for diarrhea identified included; a lack of education on hygiene practices, age of child, area of residence, maternal education, and source of water, toilet facility, disposal of waste and multiple children aged less than 5 yrs residing together. 40% (*n* = 6) of the studies utilized education as a key component of the interventions and it was found to be significant in 4 of those studies. A key factor which determined the child's access to safe water sources and improved sanitation and hygiene infrastructure was socioeconomic status [21]. Individuals with higher socioeconomic index especially in the middle 40% generally reflected the positive outcomes of the health interventions compared to those with a lower index [18]. The incidence of respiratory infections was also significantly associated with independent variables including child's age, gender, and family wealth [23].

### 6.5. Adoption of Point-of-Use Water Treatment in Schools

Only 2 of the studies reviewed assessed adoption of point of use water treatment. The studies were conducted in India, (*n* = 1) and Kenya, (*n* = 1). The study conducted in India assessed the impact of a school-based safe water intervention on household adoption of point-of-use water treatment practices while the study conducted in Kenya evaluated the role of school children in the promotion of point-of-use water treatment and hand washing in households as well as its effect on students absence. They were both randomized controlled trials and utilized combined interventions including water treatment, hygiene practices, and hygiene education. The study conducted in India did not show statistically significant results while that conducted in Kenya showed improved knowledge of water treatment (49–91%, *P* < 0.0001) and this effect was retained even after a followup period of 13 months (*P* = 0.53). Absenteeism rates were also significantly reduced by about 26% [28].

## 7. Discussion

Results of our systematic review yielded fifteen studies which assessed the impact of WASH practices to improve the knowledge, attitudes, practices, and child health outcomes. These health outcomes include absenteeism, diarrhea, acute respiratory infections, and adoption of point of use water treatment. More than half of the studies (53%, *n* = 8) were randomized controlled trials, followed by 40% (*n* = 6) cross-sectional studies and 6% (*n* = 1) being a case series study. 60% (*n* = 9) of the studies were interventions that involved water treatment, hygiene practices, hygiene education, and sanitation in single form or in combination. Various data collected included socio-demographics, household and school characteristics, and environmental factors.

Higher rates of infection by helminthes and protozoa were more prevalent in the younger age group consisting of children aged 7-8 years old compared to the older children aged 8 to 10 years [19]. Negative child health outcomes were more common in children in lower grade levels, specifically grade 3 [14]. The impact of the WASH interventions differed with respect to gender and socioeconomic indices with children in higher socioeconomic levels showing better outcomes [21]. 44.4% (*n* = 4) of the studies that assessed latrine coverage showed significant association with the outcomes while 50% (*n* = 2) of the studies that assessed school area (highlands versus lowlands) and water sources found a significant association with the outcomes. The structure, cleanliness, and general outlook of latrines were implicated in the rate of utilization of sanitary facilities by the school children, with children being more likely to utilize well-kept sanitary facilities or outside sources [19].

The most commonly assessed variables with highest significance were latrine coverage and hand washing practices while the least commonly assessed were school characteristics including pupil-latrine ratio, availability of soaps in toilets, water source contamination, water availability, drying material availability, and several household variables including number of people sharing bedroom or toilet with child. This review showed that several independent variables were implicated in the adoption of WASH practices and found to be significantly associated with the outcomes. These variables should be carefully studied in future randomized controlled interventions. They include the age of the child, gender, grade level, socioeconomic index, access to hygiene and sanitary facilities, and prior knowledge of hygiene practices.

All the studies done in developing countries reported a positive effect of sanitation practices on reducing diarrhea prevalence [19, 22, 28]. Most of the studies involved children within grade level 3, who were at high risk for gastrointestinal and acute respiratory infections which could have been a source of bias. Studies have shown that children in grade 3 are at high risk of being infected with schistosomiasis in communities with high prevalence of this disease [23]. Some of the limitations observed in the various studies in this group included self-reporting of illness incidences leading to misclassification, underrepresented sample sizes, recall bias by parents in diarrheal incidences of their children, restricting studies to a particular age group, gender restriction, and the use of cross-sectional study designs [27]. Loss to followup of participants was also predominant. In the study done in Pittsburg, reason for absence in students could only be ascertained in 34% of absences; this is largely attributed to the inability to contact the parent or guardian [30]. Other limitations included a confounding of the impact of interventions due to similar ongoing interventions making it difficult to evaluate the outcomes, restriction of the study to a particular gender, and study sample restriction to only children present at the start of the school term leading to a loss of generalizability [13, 21, 27, 30].

## 8. Conclusion

This review identified a gap in assessment of nutrition practices which is a key factor related to the various outcomes studied especially diarrheal infections and should therefore be given more attention in future research. The studies assessed the health and educational effects of WASH practices in schools on reducing absenteeism and diarrhea prevalence/infections among school-age children on a short term. However, there have been little or no empirical studies which examined the long term impact of WASH interventions on child health outcomes, and therefore limited data to support future interventions. This was noted as a limitation in various studies showing a high loss to followup, where followup was present [30]. The positive effect of an education component in the intervention on the uptake and adherence to hygiene practices should be noted in future research. Knowledge was implicated in several studies in this review as a facilitator in the uptake of hygiene practices and interventions [5, 21]. Several key independent variables including age of the child, gender, grade level, socioeconomic index, access to hygiene and sanitary facilities use and prior knowledge of hygiene practices which were significantly associated with child health outcomes should be noted and controlled for in future interventions. The review concluded that the importance of access to safe water, hand washing facilities, and hygiene education cannot be underscored in abating water-borne illnesses, malnutrition, school absenteeism, and generally improving the quality of life and learning performance in children.

## Figures and Tables

**Table 1:** Table showing independent variables assessed.

Categories	Variables	Number of studies assessing variables	Study location		Impact on outcomes	
			Rural based	Urban based	Rural based	Urban based
Sociodemographics	Age	12	6	6	2	1
	Gender	12	7	5	1	2
	Knowledge	12	6	6	5	6
	Household income	9	5	4		3
	Grade level of children	6	3	3	2	1
	Maternal education	2	2		2	1
	Parental occupation	1		1		
	Parent education	5	4	1	3	

Household variables	Female-headed households	3	2	1	1	1
	Distance to school from home	1		1		
	Distance to water source	2	1	1		
	Family size	4	3	1	1	
	No. of people sharing bedroom with child	1		1		
	No. of people sharing toilet with child	1		1		
	No. of children <5 yrs in household	2	1	1	1	

School characteristics	School area/location (highland/lowland)	2	2		1	
	Latrine coverage	9	6	3	3	1
	Pupil-latrine ratio	1		1		
	Water source at school	2	1	1	1	
	Hand washing soap on basin	1	1			
	Means of waste disposal	1	1			
	Washup point after toilet	1	1			

Environmental and access	Water source contamination	1	1		1	
	Water samples	4	3	1		
	Chlorine testing	3	2	1		
	Water access/availability	1		1		
	Drinking water sources	7	5	2	3	
	Climatic season	2	1	1	1	

Nutrition practices	Food handling	2	1	1		
	Meat consumption/eating raw vegetables/eating orange peels	1	1			
	Acute malnutrition	1		1		
	Stool samples	1	1			

Knowledge, attitudes, and practices	Prior knowledge of hygiene practices and water treatment	7	6	1	4	1
	Use of soap	7	3	4	3	4
	Latrine coverage (school/household)	9	6	3	3	1
	Hand washing	10	6	4	4	4
	Sanitation practice (latrine use)	8	5	3	5	3
	Drying material availability	1		1		1
	Household water treatment	2	2		2	
	Water storage practices	3	3			
	Hygiene practices	3	2	1	2	1

**Table 2:** Description of the outcomes assessed and interventions utilized.

	Interventions	Locations	Results/significance	Grade level
Absenteeism	Hygiene education, hygiene practices, and access to sanitation facilities	Cambodia, Kenya, US, Egypt, and Denmark	Yes	1–8

Infections (diarrhea, respiratory, and gastrointestinal)	Hygiene education, water treatment, and access to hygiene resources such as installation of water stations and provision of hand sanitizers	US, Egypt, Denmark, and Zimbabwe	Yes	1–8

Adoption of point of use water treatment	Water treatment and education on hygiene practices	India	Yes (not statistically significant)	Not specified
